# Volar fixed plating of distal radius fractures: optimizing plate position for enhanced clinical outcomes

**DOI:** 10.1186/s12891-024-07415-z

**Published:** 2024-04-23

**Authors:** Abdulsamet Emet, Enejd Veizi, Yavuz Karaman, Erkan Akgun, Tolga Tolunay, Ahmet Firat

**Affiliations:** 1 Department of Orthopedics and Traumatology, Etlik City Hospital, Turan Gunes Blv. Koz Apt. 41/22 Cankaya, Ankara, Turkey; 2 Ankara City Hospital, Department of Orthopedics and Traumatology, Yıldırım Beyazıt University, Ankara, Turkey; 3 Department of Orthopedics and Traumatology, Ankara City Hospital, Ankara, Turkey; 4 Department of Orthopedics and Traumatology, Gazi University, Ankara, Turkey

**Keywords:** Distal radius, Volar plate, Plate position, Wrist, Radius fracture

## Abstract

**Background:**

The precise influence of plate position on clinical outcomes in the context of volar fixed-angle plating for distal radius fractures is not fully understood. This article aims to investigate the influence of plate position on clinical results, and functional outcomes in patients treated with volar fixed plating for distal radius fractures.

**Methods:**

A total of 58 patients with 64 distal radius fractures were included in the study. Patient demographics, fracture characteristics, surgical details, and radiographic data were collected. Post-operative AP and Lat views of all patients taken on the first day after surgery were evaluated. Volar Tilt, Radial Inclination and Radial Height measurements were used as reduction criteria. In the follow-up, the patients were called for their last control, flexion and extension angles of the wrist and Mayo Wrist Scores, the distance of the plate to the joint line and the angle between the plate and the radial shaft were measured and recorded.

**Results:**

A total of 64 distal radius fractures, with a mean age of 46.9 years, and the mean follow-up period 24.9 months were included in this study. There was a significant relationship between the Radial Inclination and Plate-Shaft Angle variables and the Mayo Wrist Score at a 99% confidence interval. Additionally, a relationship was observed between the Radial Height variable and the Mayo Score at a 90% confidence interval. A significant positive association was observed between radial inclination and achieving a Good-to-Excellent Mayo score (OR = 1.28, 95% CI [1.08–1.51], *p* = 0.004). Plate distance to joint line demonstrated a marginally significant positive association with a Good-to-Excellent Mayo score (OR = 1.31, 95% CI [0.97–1.77], *p* = 0.077). Univariate analysis revealed a significant negative association between plate-shaft angle and achieving a Good-to-Excellent Mayo score (OR = 0.71, 95% CI [0.52–0.99], *p* = 0.045). This negative association remained statistically significant in the multivariate analysis (*p* = 0.016).

**Conclusion:**

Radial inclination, plate distance to joint line, and angle between plate and radius shaft were identified as significant factors associated with improved Mayo Wrist Scores.

## Introduction

Distal radius fractures (DRFs) account for a significant proportion of traumatic injuries and are a frequent cause of disability, affecting individuals of all ages [1]. The management of DRFs has evolved substantially over the years. Conservative treatment has historically been the mainstay for uncomplicated cases; however, surgical interventions have become increasingly favored, particularly in patients with displaced, intra-articular, or comminuted fractures [2–4]. Among these, volar fixed plating has gained popularity as a reliable and effective method to stabilize and restore the distal radius anatomy [5].

The primary objective of plate fixation is to achieve anatomical reduction of the fractured fragments, restoring the congruity of the distal radioulnar joint and the radiocarpal joint [6]. To accomplish this, orthopedic surgeons face a challenging task. Traditionally, fracture classification and anatomical reduction have been measured using standard X-rays. Many subsequent studies have examined the association of pre- and post-reduction radiographic measures with functional or patient-reported outcomes [7, 8]. However, controversy exists in the literature surrounding the impact of radiographic measures on predictors of patient outcomes. A cohort of patients over 50 years reported that acceptable radiographic reduction was not associated with better physical or mental health status, lower disability, or greater satisfaction [9].

In displaced distal radius fractures, regaining and restoring alignment is important for good clinical outcomes [4]. Though, the success of volar fixed plating is closely linked to the precise placement of the plate. Achieving optimal plate position is crucial in maintaining fracture reduction and minimizing complications that can impact patient recovery. Plate malposition may lead to joint incongruity, decreased wrist motion, and implant-related tendon irritation hindering the attainment of optimal clinical outcomes [10].

There is no consensus on which radiographic parameter is the best predictor for deciding on the outcome of surgical treatment [11]. Despite the widespread use of volar fixed plating, the optimal plate position and its direct influence on clinical outcomes remain areas of ongoing research and debate [12, 13]. Numerous studies have investigated the importance of plate positioning in the context of distal radius fractures, but a comprehensive analysis is still warranted to unveil the complex interplay between plate position and various clinical parameters. This study aims to investigate the relationship between radiographic and clinical outcomes in patients treated with volar fixed plating for DRFs, specifically, the interplay between plate position and radiographic reduction parameters and functional results, shedding light on the influence of plate positioning on functional recovery and patient satisfaction.

## Materials and methods

This study employed a retrospective observational design to analyze the outcomes of volar fixed plating in the treatment of distal radius fractures. The study was initiated after the ethics committee approval was obtained from the Ankara City Hospital Ethics Committee (Ankara City Hospital, E2-23-3726). Written informed consent was obtained from each patient. Patients admitted to the Adult Emergency Clinic with the diagnosis of distal radius fracture between January 2018 and December 2022 are listed. Patient demographics, fracture characteristics, surgical details, and radiographic data were extracted from electronic medical records. Fracture patterns were classified according to the AO/OTA classification system, and associated injuries were documented. Preoperative radiographs, computed tomography (CT) scans, and/or magnetic resonance imaging (MRI) were reviewed to assess fracture morphology, displacement, and comminution. A total of 58 patients with 64 distal radius fractures were included in the study. Patients with tendon injury during distal radius fracture, open fractures, patients with neurological deficits in the upper extremity to be operated on, and patients treated other than volar locking plate were excluded from the study. All patients who underwent open reduction with the Modified Henry approach and implanted volar locking plate (Micron A.S., Ankara, Turkey) in the Orthopedics and Traumatology Clinic and were able to come to the final follow-up were included in the study. All patients were operated by same surgical team.

### Operative details

An incision was made overlaying the FCR (Flexor Carpi Radialis) tendon. The interval of the dissection proceeded between the FCR and the Radial Artery. After FCR tendon sheath is incised sharply in a longitudinal direction, blunt finger dissection was applied, flexor pollicis longus muscle was swept in an ulnar direction, exposing the underlying pronator quadratus. The pronator was released from the radius with an inverted L-shaped incision, and the muscle is dissected away from the underlying periosteum of the distal radius. After applying of open reduction and volar plate fixation, all patients had pronator quadratus repair. Short arm splint applied after the layers of the patients are closed anatomically. The patients were called for control one week after the operation and the physical therapy program was started and the same program was applied to each patient.

### Radiographic evaluation

**Fig. 1:**
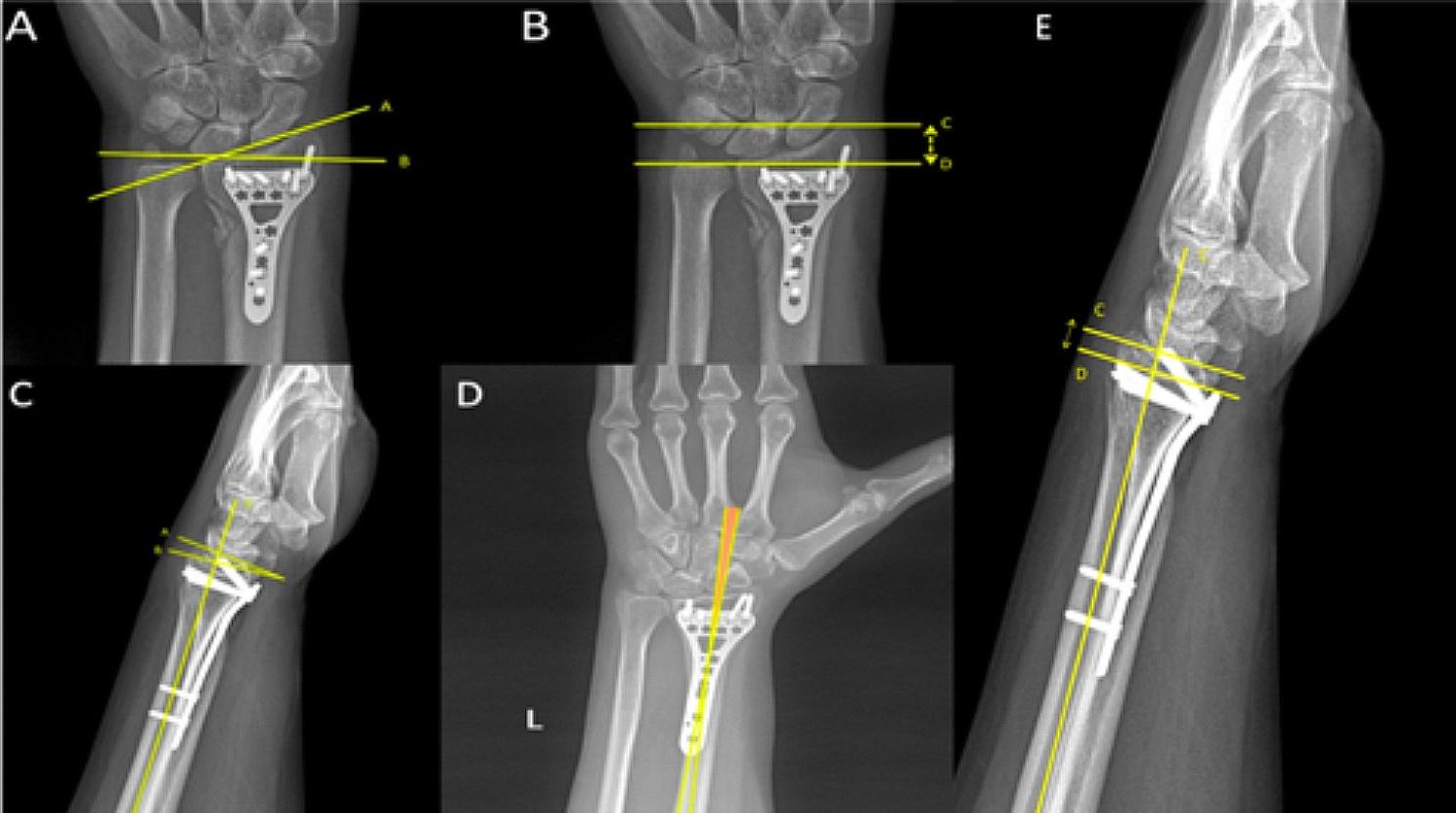
Measurements (**A**) Radial inclination (**B**) Radial height (**C**) Volar tilt (**D**) Plate-shaft angle to Radius shaft (**E**) Plate distance to joint line

X-rays were taken in the Department of Radiology under the supervision of an orthopedic surgeon in a single X-ray device. The recordings were made by keeping the tube at a distance of 100 cm from the limb. X-rays were evaluated over the measurement section in the PACS (Centricity, General Electric Health Systems, Waukesha, WI, USA) system. The distance of the plate to the joint line was measured by two different observers from the lateral x-ray and the plate radial shaft angle was measured by two different observers from the AP view (Fig. 1).

### Data collection

Postoperative radiographs were assessed to determine the accuracy of plate positioning and fracture reduction. Post-operative AP and Lat views of all patients taken on the first day after surgery were evaluated retrospectively. Volar Tilt, Radial Inclination and Radial Height measurements were used as reduction criteria. In the follow-up, the patients were called for their last control, flexion and extension angles of the wrist and Mayo Wrist Scores were recorded during the examination. In the final control x-ray, the distance of the plate to the joint line and the angle between the plate and the radial shaft were measured and recorded.

### Statistical analysis

Statistical analysis was performed in the Statistical Package for the Social Sciences (SPSS) 22 package program. Descriptive statistical results are presented as medians (minimum-maximum) and frequencies with percentages. For normality analysis, it was investigated whether skewness and kurtosis values were within the normality test limits (-/+ 2) for the subject series. The analyses were the Mann-Whitney U-test, chi-square test, and Fisher’s exact test. The correlations and significance levels between the variables were found using the Pearson Correlation Method. Predictors for Good to Excellent Mayo Wrist Score were analyzed using univariate and multivariate logistic regression analysis. The results had a 95% confidence interval, and the statistical significance was at the *p* < 0.05 level.

## Results

A total of 64 distal radius fractures were included in this study, with a mean age of 46.9 years (SD = 15.9). The age range varied from 18 to 65 years, with a median age of 46 years (range: 18–65). Regarding the side of the fracture, the distribution was fairly balanced, with 30 patients (46.9%) presenting with fractures on the left side and 34 patients (53.1%) on the right side. The mean follow-up period for patients in this study was 24.9 months (SD = 14.4), with a median follow-up duration of 21.5 months (range: 12.0–59.0). The distribution of fracture types according to the AO classification was shown at Table 1.

**Table 1:** Demographic characteristics of the study cohort

	*n* = 64
**Age**	
Mean ± SD	46.9 ± 15.9
Median (min – max)	46 (18–65)
**Side**	
Left	30 (46.9%)
Right	34 (53.1%)
**Fracture type (AO)**	
23A2	10 (15.6%)
23A3	1 (1.6%)
23B1	1 (1.6%)
23B2	12 (18.8%)
23B3	10 (15.6%)
23C1	1 (1.6%)
23C2	21 (32.8%)
23C3	8 (12.5%)
**Follow up period**	
Mean ± SD	24.9 ± 14.4
Median (min – max)	21.5 (12.0–59.0)

The overall measurements and scores for a sample of 64 individuals’ are given in Table 2.

**Table 2:** Overall clinical and radiological results of the study cohort on the last follow-up

*n* = 64	Mean ± SD Median (min – max)
Extension	49.8 ± 16.8 45 (20–90)
Flexion	80.2 ± 22.6 80 (30–130)
Mayo wrist score	79.4 ± 15.2 80 (50–100)
Radial inclination	18.3 ± 4.6 18.4 (1.6–29.5)
Radial height	11.1 ± 3.2 10.2 (4.2–20.7)
Volar tilt	8.4 ± 6.0 9.0 (-11.1–21.4)
Plate distance to joint line	3.6 ± 1.7 3.5 (0.0–8.0)
Plate-shaft angle	3.1 ± 1.7 2.8 (0.4–8.1)

To analyze the direction and strength of the relationship between the Mayo Wrist Score and radiological variables, a Pearson Correlation test was conducted in accordance with the normality condition. According to the test results, there was a significant relationship between the Radial Inclination and Plate-Shaft Angle variables and the Mayo Wrist Score at a 99% confidence interval. Additionally, a relationship was observed between the Radial Height variable and the Mayo Score at a 90% confidence interval.

Specifically, a weak positive relationship was found between Radial Inclination and the Mayo Wrist Score, a weak negative relationship between Plate-Shaft Angle and the Mayo Wrist Score, and a very weak positive relationship between Radial Height and the Mayo Wrist Score.

No significant relationship was detected between the Mayo Score and other variables (Fracture Type, Volar Tilt, and Plate distance to joint line) (Table 3).

**Table 3:** Correlation analysis of the radiological variables and the overall mayo wrist score

		Fracture type	Radial inclination	Radial height	Volar tilt	Plate distance to joint line	Plate-shaft angle
**Mayo Wrist score**	Pearson Correlation	-,141	,455^***^	,222^*^	-,077	,094	-,372^***^
	Sig. (2-tailed)	,268	,000	,078	,545	,462	,002
	N	64	64	64	64	64	64

*** Correlation is significant at the 0.01 level (2-tailed)

** Correlation is significant at the 0.05 level (2-tailed)

* Correlation is significant at the 0.10 level (2-tailed)

When the results of Table 4 are evaluated, a weak positive relationship at a 95% confidence level was found between Flexion and Extension; a moderately positive relationship at a 99% confidence level was observed between the Mayo Score and Flexion, while a weak negative relationship at a 95% confidence level was detected between the Mayo Score and Plate-shaft angle. No statistically significant relationship was found between Flexion and Plate distance to joint line. A moderately positive relationship at a 99% confidence level was found between Extension and the Mayo Score, while a weak negative relationship at a 99% confidence level was detected between Extension and Plate-shaft angle. No statistically significant relationship was found between Extension and Plate distance to joint line. A weak negative relationship at a 99% confidence level was observed between the Mayo Score and Plate-shaft angle. No statistically significant relationship was found between the Mayo Score and Plate distance to joint line. The relationship between Plate distance to joint line and all other variables was found to be statistically insignificant.

**Table 4:** Correlations and significance levels between flexion, extension, mayo wrist score and plate distance to joint line (mm), plate-shaft angle variables

		Flexion	Extension	Mayo wrist score	Plate distance to joint line	Plate-shaft angle
**Flexion**	Pearson Correlation	1	,295**	,655***	,062	-,285**
	Sig. (2-tailed)		,018	,000	,629	,023
**Extension**	Pearson Correlation	,295**	1	,501***	,026	-,401***
	Sig. (2-tailed)	,018		,000	,840	,001
**Mayo Wrist Score**	Pearson Correlation	,655^***^	,501^***^	1	,094	-,372^***^
	Sig. (2-tailed)	,000	,000		,462	,002
**Plate distance to joint line**	Pearson Correlation	,062	,026	,094	1	-,149
	Sig. (2-tailed)	,629	,840	,462		,240
**Plate-shaft angle**	Pearson Correlation	-,285**	-,401***	-,372***	-,149	1
	Sig. (2-tailed)	,023	,001	,002	,240	

*** Correlation is significant at the 0.01 level (2-tailed)

** Correlation is significant at the 0.05 level (2-tailed)

* Correlation is significant at the 0.10 level (2-tailed)

In the univariate analysis, a significant positive association was observed between radial inclination and achieving a Good-to-Excellent Mayo score (OR = 1.28, 95% CI [1.08–1.51], *p* = 0.004). This relationship remained statistically significant in the multivariate analysis after adjusting for other variables (Adjusted OR = 1.41, 95% CI [1.08–1.84], *p* = 0.011). In the univariate analysis, plate distance to joint line demonstrated a marginally significant positive association with a Good-to-Excellent Mayo score (OR = 1.31, 95% CI [0.97–1.77], *p* = 0.077). In the multivariate analysis, this association became statistically significant after adjusting for other variables (Adjusted OR = 1.60, 95% CI [1.05–2.45], *p* = 0.028). Univariate analysis revealed a significant negative association between plate-shaft angle and achieving a Good-to-Excellent Mayo score (OR = 0.71, 95% CI [0.52–0.99], *p* = 0.045). This negative association remained statistically significant in the multivariate analysis (Adjusted OR = 0.59, 95% CI [0.39–0.90], *p* = 0.016). These results suggest that radial inclination and plate-shaft angle are important factors associated with the likelihood of achieving a Good-to-Excellent Mayo score, with radial inclination showing a positive association and plate-shaft angle showing a negative association. Plate distance to joint line showed significance in the multivariate analysis, while radial height and volar tilt did not appear to significantly impact the outcome (Table 5).

**Table 5:** Regression analysis of the radiological variables and a good-to-excellent mayo wrist score

	Good-to-excellent mayo score (score > 80)				
	Univariate analysis		Multivariate analysis model		
	OR (95% CI)	***p-value***		Adjusted OR (95% CI)	***p-value***
Radial inclination	**1.28 (1.08–1.51)**	**0.004**	**Radial inclination**	**1.41 (1.08–1.84)**	**0.011**
Radial height	**1.21 (1.01–1.45)**	**0.037**	Radial height	1.17 (0.84–1.64)	0.350
Volar tilt	0.98 (0.91–1.07)	0.757	Volar tilt		
Plate distance to joint line	1.31 (0.97–1.77)	0.077	**Plate distance to joint line**	**1.60 (1.05–2.45)**	**0.028**
Plate-shaft angle	**0.71 (0.52–0.99)**	**0.045**	**Plate-shaft angle**	**0.59 (0.39–0.90)**	**0.016**

## Discussion

In the current study, we present that radial inclination, plate distance to joint line, and angle between plate and radius shaft were identified as significant factors associated with improved Mayo Wrist Scores.

Malunion is the most common complication of nonsurgical treatment of distal radius fractures and is a very common clinical entity [14]. In a recent long-term follow-up study, patients who sustained a distal radius fracture and developed malunion between the ages of 18 and 65 were more likely to have adverse clinical outcomes such as activity limitation and pain [15]. To prevent malunion, all surgical interventions aim to achieve anatomical reduction and stability of the fracture and have been shown to produce good outcomes. Locking plates are considered to provide a strong fixation with multiple papers showing good outcomes [16, 17].

Internal fixation with volar plates has seen a steady increase in use in recent years due to purported faster functional recovery and often improved radiographic alignment [18, 19]. However, with this widespread use, increasing complications have begun to be reported and these complications have been associated with plate positions [20, 21]. After evaluating the latest literature, the currently discussed issue is the relationship between volar tilt and the flexor pollicis longus (FPL) tendon in patients who have undergone surgical treatment with volar fixed plates is a topic of growing interest [22]. Chronic irritation due to altered volar tilt can lead to tendon attrition, resulting in decreased FPL tendon function over time. In a recent biomechanical study, significant increases in contact force were also observed which failed to restore the volar tilt with placement distal to the watershed line [23]. In our own study, we found that volar tilt does not affect the overall outcome, but will continue to be an important parameter among the reduction criteria.

When the literature is evaluated, there are still ongoing discussions about the watershed line [24, 25]. Many anatomical and cadaveric studies attempt to describe the exact watershed area [6, 26]. However, these ongoing discussions have still not fully defined this line. Intraoperative evaluation, a plate placed distal to this line causes serious complications [22, 27, 28]. In the light of this information, we can say that easier anatomical or radiological points are needed for intraoperative evaluation for plate positioning. In this study, it was found that distance of the plate to the joint line did not affect the reduction scoring. We concluded that the further and proximal the plate is placed from the joint, the better the clinical results since it is difficult to find the watershed line intraoperatively.

According to Rikli’s column theory, the radial styloid is an important part of the lateral column that participates in the radial height and inclination [29]. The column acts as a support to resist radial carpal translation and functions as a load-bearing platform. In addition, it serves as an anchor for the radioscaphocapitate ligament, preventing ulnar translation of the wrist [30, 31]. In addition, there are studies in the literature showing that grip strength decreases due to decreased inclination, loss of grip resulting from abnormal inclination of the articular surface of the radius is due to a dysfunction in flexor-extensor synergy [32]. It has been shown that cosmetic concern occurs with a decrease in radial inclination post-surgery or in patients followed conservatively [33]. As we know, one of the four components of the Mayo wrist score is grip strength and the second is patient satisfaction. In light of this information, as stated in many articles in the literature, reduction of the distal radial fragment during the operation is very important. In our study, we concluded that radial inclination is an important parameter, consistent with the current literature.

As far as we have researched in the English literature, this study is the first to examine the results of the plate-shaft angle in distal radius fractures treated with a volar plate. The plate-shaft angle is the angle formed between the longitudinal axis of the radius shaft and the orientation of the volar plate. Considering that the plate design is fully anatomical, there is a possibility that anatomical reduction cannot be made completely in the volar plate that is inclined to the shaft. Achieving the correct plate shaft angle is essential for restoring normal anatomy, and ensuring proper alignment of the fracture. Considering this, we can speculate that, while the plate placed at the appropriate plate-shaft angle provides additional assistance in maintaining normal anatomy, that affects the clinical outcomes of patients along with appropriate anatomical reduction. The negative association between the plate-shaft angle and Mayo Wrist scoring we found in our study led us to this idea. However, more detailed research is still needed.

Our study has limitations such as being a retrospective study and its limited ability to establish causality. Additionally, the study included a relatively small sample size; A larger sample size would increase the statistical power and generalizability of the findings. Third, the study included fractures classified using the AO/OTA system, covering a wide range of fracture types. Variability in fracture patterns can cause heterogeneity in results. Finally, the study focused primarily on plate position as a factor affecting clinical outcomes. Other important variables, such as patient comorbidities and postoperative rehabilitation, have not been fully investigated and could potentially confound the results.

In conclusion, this study provides valuable insights into the surgical management of DRFs and its impact on clinical outcomes. Plate distance to joint line, and angle between plate and radius shaft were identified as significant factors associated with improved Mayo Wrist Scores and the likelihood of achieving a Good-to-Excellent score. We predict that the distance of the plate to the joint line can be used, especially when the watershed line cannot be fully evaluated intraoperatively. The reduction scores also played a pivotal role in predicting better clinical outcomes. These findings emphasize the importance of precise reduction and careful plate positioning in the treatment of DRFs. Further research and clinical attention to these factors may contribute to enhanced patient recovery and satisfaction following volar fixed plating for DRFs.

## Data Availability

The data used and/or analyzed during the current study are available from the corresponding author on reasonable request.
